# Optimal Design to Improve the Performance of Impact Resistance and Obstacle Surmounting for Legged Robots

**DOI:** 10.3390/biomimetics11040263

**Published:** 2026-04-10

**Authors:** Jiaxu Han, Jingfu Zhao, Yue Zhu, Zhibin Song

**Affiliations:** 1 School of Mechanical Engineering, Tianjin University, Tianjin 300350, China; 2022201078@tju.edu.cn (J.Z.); 2024201057@tju.edu.cn (Y.Z.); songzhibin@tju.edu.cn (Z.S.); 2 Key Laboratory of Mechanism Theory and Equipment Design of Ministry of Education, Tianjin University, Tianjin 300350, China

**Keywords:** legged robots, optimal design, impact resistance, obstacle surmounting

## Abstract

Legged robots are widely used for walking, running, jumping, and landing on the ground. As mission terrains become increasingly complex, legged robots with greater adaptability are required. However, limited research attention has been paid to enhancing their impact resistance and obstacle-surmounting capabilities. Due to the limitations of motor manufacturing and material, it is more difficult to improve the impact resistance of the motor than to design proper leg lengths. Considering rigid multi-link medium- and large-sized legged robots, we optimize leg lengths to minimize the impact torque on leg joints. An optimal leg-length combination that maximizes obstacle-surmounting capability for medium- and large-size multi-link legged robots is conducted. This research provides a concrete design basis for leg-length optimization in medium- and large-sized multi-link legged robots with the aim of improving impact resistance and obstacle surmounting.

## 1. Introduction

Legged robots with link structures have gained significant attention in the field of robotics due to their versatility and adaptability across various terrains and applications [1]. Researchers in the field of micro-actuator systems have provided new ideas for improving system power density through folding mechanisms made of deformable metamaterials [2]. This approach may find future applications in buffering that involves storing and releasing energy under impact. The bio-inspired experts in artificial intelligence and multi-functional material use novel hardware in legged applications to improve memory capability and power efficiency with neuromorphic electronics, such as synaptic ion-gating vertical transistors [3]. Neuromorphic organization, computational units, and hardware converge in biology-first controllers to achieve mobility rivaling that of their biological counterparts; there is a need to apply more biomimetic solutions to unsolved problems in robotics [4]. The research above helps bridge the gap regarding the optimization and miniaturization of legged robots. There are also some challenges and difficulties in medium- and large-sized legged robots. Their abilities to surmount obstacles and resist impacts are critical factors influencing their suitability for complex environments and tasks [5]. These robots often mimic animal locomotion, and the design of their legs plays a pivotal role in determining overall performance [6]. Leg length, in particular, is a key parameter that significantly affects the kinematics and dynamics of legged robots with a multi-link structure [7]. Leg-length optimization is a complex, multidisciplinary challenge that has been the subject of extensive research [8]. Achieving an optimal leg length can lead to improvements in mobility, energy efficiency, and overall performance [9]. Numerous bio-inspired legged robots with rigid multi-link structures have been developed, including BigDog [10], MIT Cheetah [11], ANYmal [12], and HyQ [13]. Although the biomimetic approach is feasible for leg design with a multi-link structure, it may not yield the optimal solution for a given robot or task. The optimal leg length depends on the specific task. Therefore, a specialized leg design is required to ensure satisfactory performance for a particular application.

Owing to limitations in manufacturing and processing, improving the performance of the motor or material is more challenging than designing appropriate leg lengths. Numerous robotics experts have investigated leg lengths under various task scenarios [14,15,16]. Sugimoto et al. designed and analyzed the unique walking gait mechanism of a locust robot, focusing on the difference in leg lengths [17]. Their study suggested that the ground reaction forces may also vary with different leg lengths and that further research should be conducted on this aspect. Zhang et al. designed a jumping robot inspired by nature and proposed a leg-length adjustment approach to adapt to the different requirements of jumping height and distance of the jumping robot [18]. Steudel-Numbers et al. found that people with relatively longer lower limb length have lower locomotor costs during walking and running [19]. Li et al. performed the staircase-climbing capability-based dimension design of a hexapod robot and obtained the relationship between the leg dimensions and staircase-climbing capability [20]. Chadwick et al. presented an open-source framework for developing optimal leg designs for walking robots that can optimize the leg lengths and transmission ratios for a user-defined metric, such as the minimization of energy consumption, enabling the user to better navigate through the high-dimensional and unintuitive design space [21].

The ability to overcome obstacles and withstand collisions enables legged robots to adapt to diverse terrains and tasks. Such adaptability is essential for applications in environments with uneven surfaces or obstacles. However, few studies have addressed obstacle surmounting and impact resistance for legged robots through leg-length optimization. Legged robots equipped with obstacle-surmounting features can efficiently navigate obstacles, thereby improving mission efficiency in scenarios such as search and rescue operations or exploration missions. Park et al. presented a planning framework via heterogeneous simplified models for jumping over obstacles with a quadruped robot named MIT Cheetah [22]. Stasse et al. described a method to modify the feet trajectory that considers obstacle avoidance, joint limits, dynamical stability, and impact to use the robot HRP-2 to step over large obstacles [23]. Guan et al. focused on feasibility analysis and motion planning for determining whether the robot HRP-2 can step over a given obstacle and how to step over the obstacle [24].

Impact resistance is crucial for protecting the mechanical structure of legged robots. Legged robots equipped with effective impact resistance mechanisms can withstand shocks during landings or collisions, thereby ensuring the longevity and durability of the robot. Jie Chen et al. designed legged robots using compliant mechanisms in the distal segments to provide better buffering performance [25]. Yin et al. presented a novel legged robot with repetitive landing capacity that could perform soft-landing tasks repeatedly at multiple landing sites on the Moon [26]. Wang et al. developed a highly dynamic legged robot with an enhanced capability of high jumping and impact mitigation and achieved a jumping height equal to three times the robot leg length and a soft landing [27]. Yang et al. presented a leg locomotion system, including the nonlinear active compliance control and the active impedance control for the steel wire transmission-based legged robot, and enabled high-speed dynamic locomotion with excellent impact mitigation performance [28]. Cho et al. analyzed the mechanical structure of a robotic leg during running for impact mitigation [29].

Impact resistance and obstacle surmounting are two critical capabilities for legged robots, as they fundamentally determine a robot’s mobility and operational workspace. However, there is a notable scarcity of studies on leg-length optimization that consider the capabilities of impact resistance and obstacle surmounting. Therefore, it is necessary to leverage easily adjustable leg lengths to enhance the obstacle surmounting and impact resistance capabilities of legged robots. In this study, an optimization design solution to improve the performance of impact resistance and obstacle surmounting for rigid multi-link structure medium- and large-size legged robots is proposed. The optimization variables in this paper towards the rigid multi-link structure legged robot are based on the easily adjustable leg-length relationships, as different leg structures vary in design. The leg length is the fundamental and easily modifiable structural component of such legged robots. Consequently, the optimization design method proposed in this paper holds significant value for optimizing various types of robot legs.

## 2. Methods and Results

### 2.1. Design of the Legged Robot

**Figure 1:**
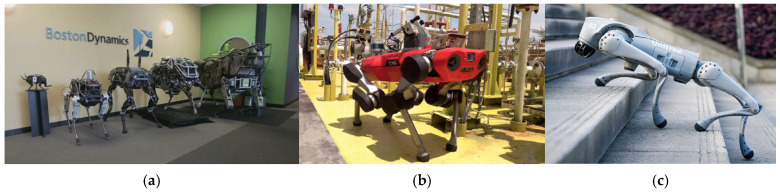
Legged robot with multi-link mechanism contained e (**a**) ANYmal by ETH Zurich (Zurich, Switzerland); (**b**) Big Dog by MIT and Boston Dynamics (MA, USA); (**c**) Go1 by Unitree (Hangzhou, China).

The linkage mechanisms have the advantages of simple structure, high transmission efficiency, and high load capacity. Many researchers design legged robots used the linkages mechanisms, such as Big Dog (MA, USA), MIT Cheetah (MA, USA), Spot mini (MA, USA), StarlETH (Zurich, Switzerland), ANYmal (Zurich, Switzerland), Go1 (Hangzhou, China), and HyQ (Genoa, Italy). The optimization of leg-length relationships in this paper is aimed at medium- and large-scale rigid multi-link legged robots, such as the legged robot shown in Figure 1.

**Figure 2:**
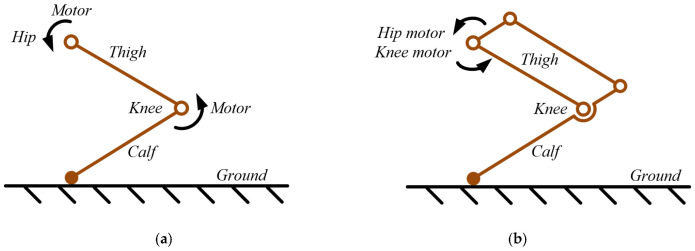
Schematic diagram of the legged robot, showing (**a**) traditional structure and (**b**) with a parallelogram mechanism.

For legged robots of such scale, the leg structure is made of ultra-light materials, making the leg mass far smaller than that of the motors and body. As a result, changes in leg-length relationships have little effect on mass distribution. The legged robot mechanism is simplified to the linkage mechanism as shown in Figure 2a. The legged robot is essentially composed of a thigh, calf, hip joint, and knee joint. Motors are mounted separately at the knee and hip joints to drive the thigh and calf, respectively. In order to reduce the inertia and mass of the legs, the knee motor that was originally positioned between the thigh and shin has been relocated to the hip joint through the parallelogram mechanism, as shown in Figure 2b. The thigh and shank no longer house any motors, sensors, or wires. These components are now positioned at the hip joint and on the legged robot body. Therefore, the variation in leg length does not affect the placement of sensors, wires, and motors. Using the optimization method to investigate the relationship between the above leg parameters and the performance of rigid multi-link legged robots is important for a better understanding and utilization of this type of legged robot.

### 2.2. Impact Resistance Optimization for Legged Robot

Impact upon landing may cause damage to the internal components of legged robots, leading to decreased performance or functional failure. A buffering optimization design system can protect the critical components of the robots. The motor is the most crucial and costly component that is susceptible to damage in legged robots. When a legged robot falls from a height, the impact force from the ground is transmitted to the joint motor shaft, creating an impact torque. When the impact torque is excessive, it can easily result in damage to the motor output shaft and the gear reducer. Given the magnitude of the ground force, our task is to explore how to reduce the impact torque on the motor shaft. Due to limitations in motor manufacturing and processing, the current improvement in the impact resistance of motor joints is quite limited. However, the optimization of leg-length relationships and posture at landing is relatively easier compared to enhancing motor performance.

**Figure 3:**
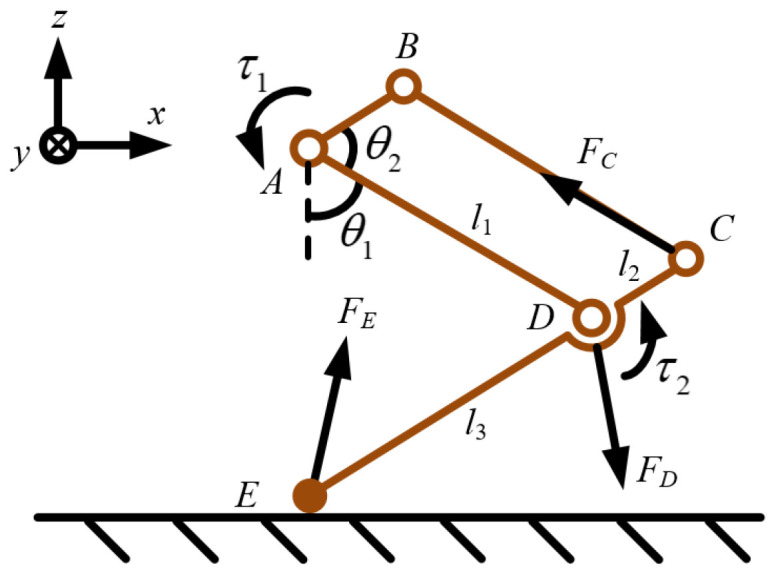
Kinematic and mechanical parameters of the legged robot.

We employ a static equilibrium approach to calculate the extremum of the dynamic impact torque. This choice is motivated by two considerations. First, modeling and estimating the dynamic transmission and energy dissipation of ground impact forces from the leg end to the motor output shaft during the instant of impact is challenging. Second, this approach is intended to better protect legged robots. In the static equilibrium state, the instantaneous maximum force during impact is greater than that in the dynamic state. From a force transmission perspective, the ground force can be maximally transmitted to the motor shaft. Under the same ground impact force, energy is dissipated in components such as links and joints in a non-static equilibrium state due to energy conservation. In contrast, static equilibrium calculations assume no energy loss during transmission. Consequently, the instantaneous maximum impact force in the static equilibrium state exceeds that in the dynamic state, making static equilibrium analysis more stringent and less problematic than dynamic analysis. To ensure structural protection, the allowable load should be less than the maximum impact load. Applying the most stringent conditions is advantageous for safeguarding legged robots. Therefore, we simplify the dynamic problem into a more rigorous static equilibrium problem for analysis. The next step is to investigate the leg-length ratios and joint angles that minimize the impact torque on the joints under a given ground impact force. The kinematic and mechanical parameters of the legged robot are shown in Figure 3.

Let *θ*_1_ and *θ*_2_ denote the joint angles of the hip joint and the knee joint, respectively. *l*_1_ and *l*_3_ represent the lengths of the thigh and the calf, respectively, while *l*_2_ represents the length of the short bar of the parallelogram mechanism. Thus, we obtain *AD* = *BC* = *l*_1_ and *AB* = *CD* = *l*_2_. Let *F_E_* be the ground contact force, *τ*_1_ and *τ*_2_ denote the impact torque of the hip joint motor and the knee joint motor, respectively.

To control variables and better explore the leg length and the location of its placement directly below, the goal is to use ground reaction forces to measure the magnitude of impact torque on the hip joint and the knee joint. In the optimization condition search, the hip joint angle and the knee joint angle, as well as the length of the leg, are randomly searched within the constraint conditions. Let *θ*(*F_E_*,*z*) denote the angle between the directions of the ground reaction force and the *z* coordinate axes. Furthermore, the angle between the long rod *EC* and the vertical direction in the triangle *ADE*, denoted as *θ*(*CE*,*z*), can be derived as follows.

(1) $$\theta (CE,z)=\pi -\theta_{1}-\theta_{2}$$

Taking the calf as the object under the condition of moment equilibrium, the angles between the force directions and the rod *CE* are

(2) $$\theta (F_{D},CE)=\theta (CE,z)+\theta (F_{D},z)$$

(3) $$\theta (F_{E},CE)=\theta (CE,z)-\theta (F_{E},z)$$

Moments are taken separately at point *C*, and point *D* can be obtained as follows.

(4) $$F_{D}\cdot l_{2}\sin\theta (F_{D},CD)=F_{E}\cdot (l_{2}+l_{3})\sin\theta (F_{E},CE)$$

(5) $$F_{C}\cdot l_{2}\sin\theta (F_{C},CD)=F_{E}\cdot l_{3}\sin\theta (F_{E},DE)$$

Since the rod *BC* is a two-force member, the direction of the force *F_C_* is the same as the direction of the rod *BC*. With all the terms in Equation (5) known, the force *F_C_* can be determined. According to the force equilibrium equation, the equations can be calculated as follows.

(6) $$F_{C}\sin\theta (F_{C},z)-F_{D}\sin\theta (F_{D},z)-F_{E}\sin\theta (F_{E},z)=0$$

(7) $$F_{C}\cos\theta (F_{C},z)-F_{D}\cos\theta (F_{D},z)+F_{E}\cos\theta (F_{E},z)=0$$

The direction of the force *F_D_* can be obtained through the force balance equation. At this point, all terms in Equation (4) are known, and the force *F_D_* can be obtained. Therefore, the torques at the hip joint *τ*_1_ and the knee joint *τ*_2_ can be determined as follows.

(8) $$\tau_{1}=F_{C}\cdot l_{2}\sin\theta (F_{C},AB)$$

(9) $$\tau_{2}=F_{D}\cdot l_{1}\sin\theta (F_{D},AD)$$

This yields the mapping relationship between the ground impact force and the electric joint impact torque. Given a ground force magnitude of 100 N, we consider various leg lengths and different ground force angle directions, and obtain the following curves through theoretical calculations and simulations.

**Figure 4:**
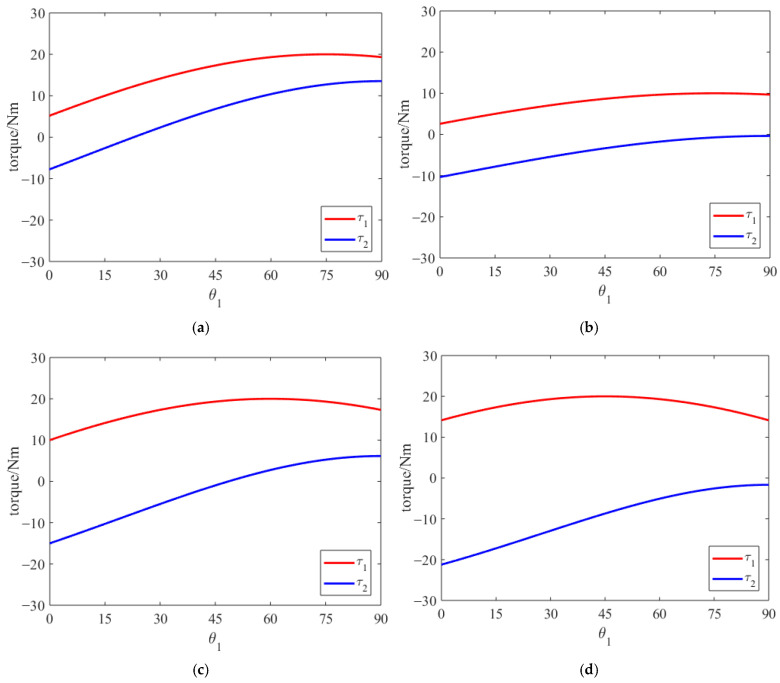
Impact torque curve of the legged robot where (**a**) *l*_1_ = 0.2 m, *l*_2_ = 0.1 m, *l*_3_ = 0.3 m, *θ*_1_ = 15°; (**b**) *l*_1_ = 0.1 m, *l*_2_ = 0.05 m, *l*_3_ = 0.4 m, *θ*_1_ = 15°; (**c**) *l*_1_ = 0.2 m, *l*_2_ = 0.1 m, *l*_3_ = 0.3 m, *θ*_1_ = 30°; (**d**) *l*_1_ = 0.2 m, *l*_2_ = 0.1 m, *l*_3_ = 0.3 m, *θ*_1_ = 45°.

The horizontal axis represents the change in knee joint angle at the moment of ground landing, and the vertical axis denotes the joint impact torque. The impact torque on the joint varies with different landing angles of the robot’s leg. From Figure 4a,b, with the same ground impact force, different leg-length parameters yield different impact torques. In Figure 4a,c,d, with the same magnitude of ground impact force but different directions, the impact torques are also different. The variation patterns of the impact torque differ under different parameters. The angles at which the impact torque reaches its extreme value are also different. Therefore, it is necessary to consider the impact torque at various landing angles.

**Figure 5:**
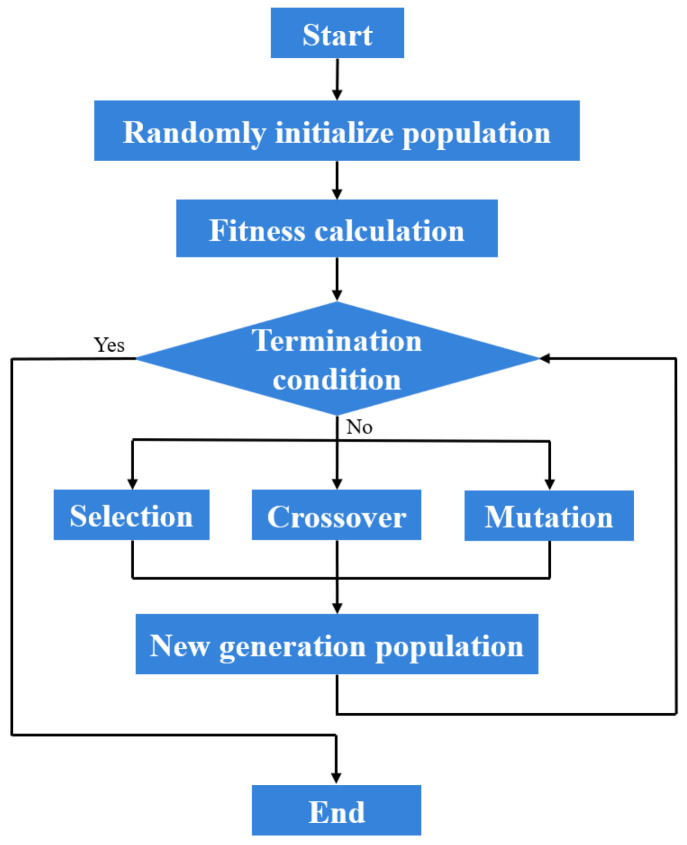
Flowchart of the genetic optimization algorithm.

Considering different combinations of leg lengths, the peak postures of impact torque vary. It is necessary to use a global stochastic optimization search method to obtain the leg-length combination that minimizes the impact torque. The classical gradient-based algorithms are prone to becoming trapped in local optima under complex optimization conditions. Consequently, modern algorithms such as genetic algorithm, particle swarm, and simulated annealing method can address this requirement. The particle swarm optimization method has a simple structure and converges quickly initially. However, the particle swarm method lacks mutation mechanisms, which makes particles prone to premature convergence. The simulated annealing method can escape local traps by accepting worse solutions, but its serial search structure leads to slow convergence. The parallel population search of the genetic algorithm, combined with crossover and mutation, ensures strong global search capability and resistance to local optima. It is naturally suited for parallel computing and demonstrates strong versatility for both continuous and discrete problems, while converging significantly faster than the simulated annealing method. The genetic algorithm is a suitable choice for our study, and the basic flowchart of the genetic algorithm is shown in Figure 5.

Develop the leg length matrix ***L***_1_ as the independent variable.

(10) $$L_{1}=\left[\begin{array}{l} l_{1}\\ l_{2}\\ l_{3} \end{array}\right]$$

Set the total length of the thigh and calf to be 0.5 m. The thigh rod length *l*_1_ is greater than or equal to the shorter parallelogram rod *l*_2_ for a compact layout, i.e., *l*_1_ ≥ *l*_2_. The lengths of both the thigh and calf are set to be greater than 0.15 m, and the length of the calf *l*_3_ is set to be greater than or equal to the length of the thigh *l*_1_, i.e., *l*_3_ ≥ *l*_1_. Therefore, the constraints for the leg-length relationships are as follows.

(11) $$\underset{L_{1}}{\min}f_{1}\left(L_{1}\right)\;s.\;t.\;\left\{\begin{array}{c} A_{1}\cdot L_{1}\leq b_{1}\\ Aeq_{1}\cdot L_{1}=beq_{1}\\ lb_{1}\leq L_{1}\leq ub_{1}\\ c_{1}\cdot L_{1}\leq 0\\ ceq_{1}\cdot L_{1}=0 \end{array}\right.$$

where

(12) $$A_{1}=\left[\begin{array}{ccc} 1 & 0 & 0\\ -1 & 0 & 0\\ 0 & -1 & 0\\ -1 & 1 & 0 \end{array}\right],b_{1}=\left[\begin{array}{c} 0.25\\ -0.15\\ -0.1\\ 0 \end{array}\right],Aeq_{1}=\left[\begin{array}{ccc} 1 & 0 & 1 \end{array}\right],beq_{1}=\left[0.5\right],$$

(13) $$lb_{1}=[\begin{array}{ccc} 0.1 & 0.1 & 0.1 \end{array}],ub_{1}=[\begin{array}{ccc} 0.5 & 0.5 & 0.5 \end{array}]$$

Let *f*_1_ denote the objective function for optimization. ***A***_1_ and ***b***_1_ represent the linear inequality constraints. ***lb***_1_ and ***ub***_1_ stand for the lower bound and the upper bound of leg lengths. To avoid excessively short rods, the initial boundary for the leg length search is set with a minimum value of 0.1 m and a maximum value of 0.5 m. ***c***_1_ represents the nonlinear inequality constraints, and ***ceq***_1_ represents the nonlinear equality constraints. Since there are no nonlinear constraints, both ***c***_1_ and ***ceq***_1_ are empty sets, i.e., ***c***_1_ = [] and ***ceq***_1_ = [].

**Figure 6:**
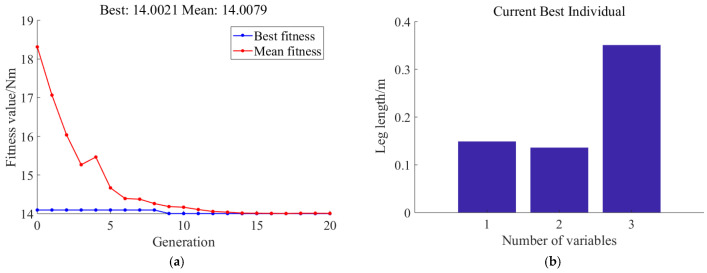
Hip joint optimization results based on the genetic algorithm contained (**a**) impact torque optimization results and (**b**) leg-length optimization results.

With the hip joint impact moment *τ*_1_ as the single-objective optimization *f*_1_, a global search for different leg-length combinations is conducted using the genetic algorithm. The optimization results obtained by minimizing the peak impact moment when landing at different angles are shown in Figure 6.

**Figure 7:**
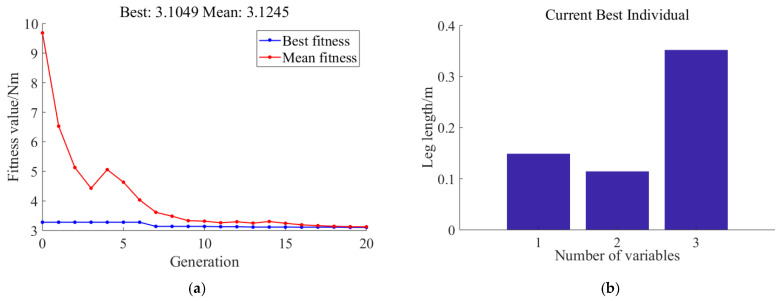
Knee joint optimization results based on the genetic algorithm contained (**a**) impact torque optimization results and (**b**) leg-length optimization results.

Through 20 iterations of particle swarm optimization, the minimum value of the maximum hip joint impact torque is found to be 14.0021 Nm. At this point, the optimal leg-length combination is a thigh length of 0.15 m, a calf length of 0.35 m, and a parallelogram leg length of 0.136 m. Using the same genetic algorithm optimization method, with the minimum value of the maximum knee joint impact torque for different leg-length combinations as the single optimization objective, the following results are obtained. Then, we set the knee joint impact moment *τ*_2_ as the optimization target, and the optimization results obtained by the genetic algorithm are shown in Figure 7.

The optimization results show that the minimum value of the maximum knee joint impact torque is 3.1049 Nm. At this point, the optimal leg-length combination is a thigh length of 0.15 m, a calf length of 0.35 m, and a parallelogram leg length of 0.14 m. Multiple iterative experiments reveal that the thigh and calf lengths converged to 0.15 m and 0.35 m, respectively. However, the optimized values of the parallelogram leg length varied significantly with each iteration, indicating that the parallelogram leg length, being a redundant constraint, has no impact on ground impact cushioning.

### 2.3. Obstacle Surmounting Optimization for Legged Robot

In legged robot design, obstacle-surmounting capability is an important criterion for evaluating the terrain adaptability of mobile robots. In some terrains, obstacles have certain heights and widths. First, it is necessary to conduct motion analysis and workspace calculation for the robot’s legs. The coordinate system and related parameters are shown in Figure 3. Taking the hip joint as the origin of the coordinate system, the *z*-axis is in the vertical direction, and the *x*-axis is in the horizontal direction. The forward kinematics equations of the legged robot are shown as follows.

(14) $$\left[\begin{array}{c} x\\ z \end{array}\right]=\left[\begin{array}{c} l_{1}\sin\theta_{1}-l_{3}\sin\left(\theta_{1}+\theta_{2}\right)\\ -l_{1}\cos\theta_{1}+l_{3}\cos\left(\theta_{1}+\theta_{2}\right) \end{array}\right]$$

**Figure 8:**
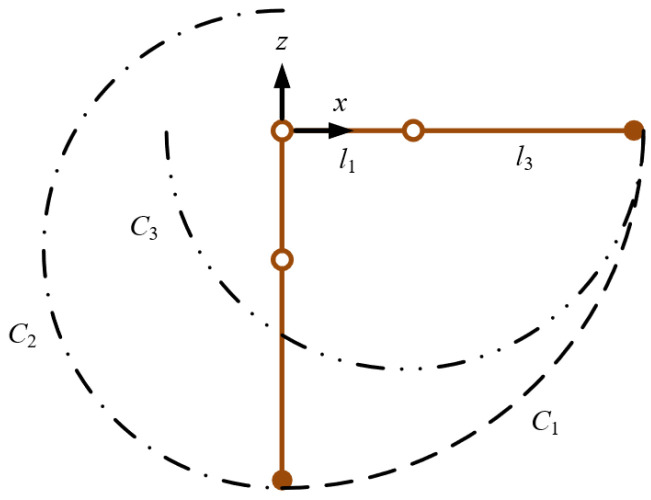
Boundary workspace of the legged robot.

The range of motion for each joint is limited due to the constraints of the mechanical structure. The angle range of the hip joint and knee joint is set to $\theta_{1}=\left[{\theta_{1}}_{\min},{\theta_{1}}_{\max}\right],\theta_{2}=\left[{\theta_{2}}_{\min},{\theta_{2}}_{\max}\right]$. Since the parallelogram mechanism is a redundant constraint mechanism, the short rod of the parallelogram *l*_2_ does not affect the kinematic characteristics. To determine the workspace of the mechanical leg, only the lengths of the thigh and calf need to be considered. Figure 8 illustrates the maximum boundary of the reachable workspace of the robot leg.

In Figure 8, the circular arc *C*_1_ represents the maximum boundary of the reachable workspace at the end of the leg. The circular arc *C*_2_ and the arc *C*_3_ denote the maximum boundaries of the calf while the thigh reaches its two limited positions. The equations for curves *C*_1_, *C*_2_, and *C*_3_ are as follows.

(15) $$x^{2}+z^{2}={\left(l_{1}+l_{3}\right)}^{2}$$

(16) $${\left(x-l_{1}\sin{\theta_{1}}_{\min}\right)}^{2}+{\left(z+l_{1}\cos{\theta_{1}}_{\min}\right)}^{2}={l_{3}}^{2}$$

(17) $${\left(x-l_{1}\sin{\theta_{1}}_{\max}\right)}^{2}+{\left(z+l_{1}\cos{\theta_{1}}_{\max}\right)}^{2}={l_{3}}^{2}$$

Arbitrarily select a point *Z*_0_ (0, *z*_0_) on the vertical coordinate as the lower boundary that satisfies the following conditions.

(18) $$-l_{1}-l_{3}<z_{0}<-l_{1}\cos{\theta_{1}}_{\max}-l_{3}$$

The coordinates of the intersection points of the horizontal plane containing *Z*_0_ with arcs *C*_1_ and *C*_2_ are $\left(\sqrt{{\left(l_{1}+l_{3}\right)}^{2}-z_{0}^{2}},z_{0}),(-\sqrt{{l_{3}}^{2}-{\left(z_{0}+l_{1}\cos{\theta_{1}}_{\min}\right)}^{2}}+l_{1}\sin{\theta_{1}}_{\min},z_{0}\right)$, respectively. In addition, take the horizontal plane at the lowest point of arc *C*_3_ as the upper boundary, with its coordinates being $\left(l_{1}\sin{\theta_{1}}_{\max},-l_{1}\cos{\theta_{1}}_{\max}-l_{3}\right)$. Therefore, the area of the rectangle enclosed by the upper and lower boundaries is

(19) $$s_{a}=\left(\sqrt{{\left(l_{1}+l_{3}\right)}^{2}-z_{0}^{2}}+\sqrt{{l_{3}}^{2}-{\left(z_{0}+l_{1}\cos{\theta_{1}}_{\min}\right)}^{2}}-l_{1}\sin{\theta_{1}}_{\min}\right)\cdot \left(-l_{1}\cos{\theta_{1}}_{\max}-l_{3}-z_{0}\right)$$

We use the ***s**_a_* as the optimization objective of the genetic algorithm optimization. The optimization variables are the leg length of the thigh and the calf. In addition, point *Z*_0_ is a randomly given variable under a constraint condition. Therefore, the optimal design variables ***L***_2_ are

(20) $$L_{2}=\left[\begin{array}{l} l_{1}\\ z_{0}\\ l_{3} \end{array}\right]$$

The constraints condition for the obstacle surmounting is shown as follows.

(21) $$\underset{L_{2}}{\min}f_{2}\left(L_{2}\right)\;s.\;t.\;\left\{\begin{array}{c} A_{2}\cdot L_{2}\leq b_{2}\\ Aeq_{2}\cdot L_{2}=beq_{2}\\ lb_{2}\leq L_{2}\leq ub_{2}\\ c_{2}\cdot L_{2}\leq 0\\ ceq_{2}\cdot L_{2}=0 \end{array}\right.$$

where

(22) $$A_{2}=\left[\begin{array}{ccc} 1 & 0 & 0\\ -1 & 0 & 0\\ -1 & -1 & -1\\ 0 & 1 & 1 \end{array}\right],b_{2}=\left[\begin{array}{c} 0.25\\ -0.15\\ 0\\ 0 \end{array}\right],Aeq_{2}=\left[\begin{array}{ccc} 1 & 0 & 1 \end{array}\right],beq_{2}=\left[0.5\right],$$

(23) $$lb_{2}=[\begin{array}{ccc} 0.1 & -0.5 & 0.1 \end{array}],ub_{2}=[\begin{array}{ccc} 0.5 & -l_{3} & 0.5 \end{array}]$$

**Figure 9:**
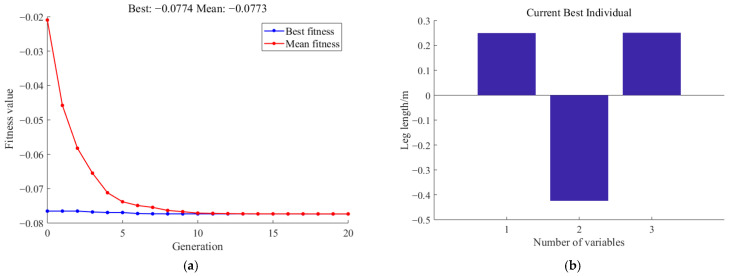
Obstacle-surmounting results based on the genetic algorithm contained (**a**) optimization process and results, and (**b**) best individual optimization results.

The optimal target is to maximize the rectangular area ***s**_a_*. Since the optimization algorithm solves for the minimum, the rectangular area ***s**_a_* is negated for the solution. In this way, the minimum value obtained through optimization corresponds to the maximum area. The optimization results obtained by the genetic algorithm are shown in Figure 9.

**Figure 10:**
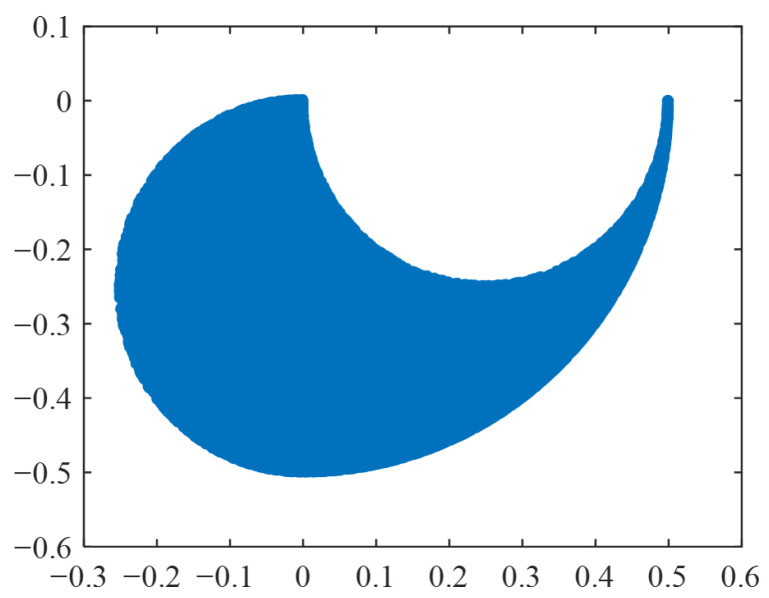
The workspace when both thigh length and calf length are 0.25 m.

Through the results, it can be observed that the area of ***s**_a_* is the largest when the lengths of the thigh and the calf are both 0.25 m, indicating that the defined obstacle-surmounting ability is the strongest. At this point, the boundary workspace of the legged robot is determined using the Monte Carlo method, as shown in Figure 10.

## 3. Discussion and Conclusions

Different leg-length combinations correspond to varying landing cushioning performance and obstacle-surmounting capabilities through the theoretical analysis. Landing cushioning ability is crucial for the safety of legged robots, while obstacle-surmounting capability determines their operational space. Focusing on these two key performance metrics, this paper investigates the relationship between leg length ratios and both landing cushioning and obstacle-surmounting performance, starting from easily adjustable leg-length combinations. Different leg-length combinations result in different peak impact moments, and the landing posture angles corresponding to these peak impact moments also vary. Therefore, this study employs a genetic algorithm to perform a global search, conducting single-objective optimization for landing cushioning and obstacle-surmounting capability separately. The optimized leg length for landing cushioning is a thigh length of 0.15 m and a shank length of 0.35 m, with an optimal landing posture at a thigh angle theta equal to 60°. The optimized leg length for obstacle-surmounting capability is a thigh length of 0.25 m and a shank length of 0.25 m. This enables designers to select leg lengths that meet their specific design requirements from the optimal results. The optimizations for landing cushioning and obstacle surmounting in this study provide a design basis for the optimized design of legged robots with a multi-link structure in medium- and large-scale environments and offer new perspectives for optimization addressing other similar application requirements. Additionally, actuator placement, torque density, transmission mechanisms, and joint loading are also promising directions for future research to improve the landing cushioning capability.

## Data Availability

The original contributions presented in this study are included in the article. Further inquiries can be directed to the corresponding author.
